# Exploring the non-linear relationship and synergistic effect between urban built environment and public sentiment integrating macro- and micro-level perspective: a case study in San Francisco

**DOI:** 10.3389/fpsyg.2024.1276923

**Published:** 2024-02-08

**Authors:** Pingge He, Bingjie Yu, Jiexi Ma, Keqian Luo, Siting Chen, Zhongwei Shen

**Affiliations:** ^1^School of Architecture, Southwest Jiaotong University, Chengdu, China; ^2^College of Architecture and Urban Planning, Chongqing Jiaotong University, Chongqing, China

**Keywords:** public sentiment, urban built environment, street visual environment, thermal comfort, non-linearity, synergistic effect

## Abstract

Public sentiment can effectively evaluate the public’s feelings of well-being in the urban environment and reflect the quality of the spatial environment to a certain extent. Previous studies on the relationship between public sentiment and urban built environmental factors have yielded meaningful results. However, few studies have focused on the effect of micro-built environment on public sentiment at the street level, which directly shapes people’s perceptions. In addition, the nonlinear relationship and synergistic effect among urban built environmental factors have been commonly disregarded in previous studies, resulting in an incomplete understanding of the impact of urban built environment on public emotions. Therefore, this paper takes San Francisco as a study case to explore the complex relationship between urban built environmental factors and public emotions. Specifically, this paper measures the polarity of public emotions through sentiment analysis on Twitter data, establishes a comprehensive built environment index system from both macro- and micro- perspectives, and subsequently explores the complex relationship between the urban built environment and public sentiment through the OLS model and Shapley Additive Explanation algorithm. Results show that: (1) micro-built environmental factors have a significant influence on public emotion, although they have been frequently ignored. (2) Public sentiment tends to be more positive in areas with recreation facilities, mixed land use, rich street view visual environment, suitable thermal and acoustic environment, balanced income, and a suitable degree of high population density. (3) A nonlinear relationship and threshold effect exist between the built environmental variables and the semantic orientations of public emotion. Environment improvement strategies based on the synergic effect between variables can effectively promote the generation of positive emotions. Our empirical findings can offer valuable insights to promote feelings of well-being and foster an urban development approach through strategic interventions within the urban built environment.

## 1 Introduction

### 1.1 Background

The urban environment has undergone significant changes due to rapid urbanization. The human perception of the urban environment has also changed over time. According to Richard Sennett, a city is not just a physical space, but a product of human interaction with the environment. This interaction shapes citizens’ identity, social relations, and sentimental cognition and influences the evolution and development of urban spatial form (Sennett, 1970). Human perception and emotion in the urban built environment can be used to measure the interaction between humans and the environment, improving the comprehensibility of urban environmental quality (Sénécal, 2007). Accordingly, understanding the relationship between public emotions and the urban environment can better reveal the dynamic urban environment from the perspective of citizens and help gain insight into how the urban environment can promote people’s happiness and well-being (Yang et al., 2022).

Emotion, as a basic motivational component of human behavior, is the result of the coordination of various factors (including human physiological characteristics, cultural background, and growth environment; Tsuchiya and Adolphs, 2006), and it fluctuates with a variety of factors, such as social, environmental, and perception (Huai and Van De Voorde, 2022). Research on the relationship between emotion and space can be traced back to the 1950s. Byrne pointed out that space can evoke emotions, and the impact of space on emotions can vary over time (Byrne, 1958). Kevin Lynch proposed the concept of “mental map,” which is an abstract spatial representation of a specific environment (space) formed in the mind of an individual based on their perception and awareness. It reflects the perceived and cognitive representation of environmental space by the human brain (Lynch, 1962). Based on this framework, Brian Goodey analyzed people’s perceptions of the urban center (i.e., the environmental space in their mind) based on the individual experience and information fragments in their mind, and furtherly established a weighted mental map to clarify people’s environmental preferences (Goodey, 1974). However, this theory only focused on subjective feelings of people and disregarded the interaction between space and people. Researchers have highlighted that while space can evoke rich human emotions, human emotions can also help improve the urban environment and the quality of space (Li et al., 2018). Therefore, clarifying the complex relationship between public emotions and the urban built environment will provide meaningful reference for urban planners and policy makers when making urban development and renewal decisions.

### 1.2 Literature review

Anderson and Smith (2001) introduced the concept of emotional geography which focuses on the relationship between emotions and geographical spaces (Anderson and Smith, 2001), marking the shift of research on human perception and urban environment from purely subjective psychological domains toward a broader social space dimension. Since then, many studies have explored the interaction between urban spaces and public emotions and accumulated meaningful results. Previous studies have demonstrated that the urban form, landscape, and climate environment have significant influences on public emotions (Leyden et al., 2011; Mouratidis and Hassan, 2020). For example, researchers observed that good spatial accessibility (Lai and Deal, 2022; Sun et al., 2023), ample and high-quality urban greenery (Huai and Van De Voorde, 2022), and a good visibility of blue and green spaces (Qiang et al., 2019) in the city can help trigger people’s positive emotions. In contrast, unfavorable climate conditions, such as drought, heat waves, and heat island, can lead to negative public emotions (Fritze et al., 2008; Holly et al., 2015). Nevertheless, researchers found that the relationship between the urban environment and public emotions is not comprehensively consistent. For example, many studies have reported that a high building density tends to evoke negative emotion (He et al., 2022) However, J. Huang et al. observed that the high building density in Hong Kong is positively correlated with urban functional diversity and can promote positive emotions among residents (Huang J. et al., 2023).

In addition, the majority of existing studies have focused on the relationship between the built environment and public emotions from a macro-perspective at the city level. However, establishing a sound and adequate indicator system for the built environment at the macro-city level is challenging for the complexity of a city. Yang et al. (2022) summarized the environmental factors affecting public emotions into four types: objective, perceived, physical, and social environments. However, which type is the most significant is obscured. How the environmental indicators at the macrolevel affect public emotions in the long term also remains unclear. Liu et al. found that the distance to the urban center have a negative impact on public emotions (Liu et al., 2020). Nevertheless, it remains unclear whether the impact is direct or indirect because the multiple collinearities and synergies might exist between the distance and other factors such as income, quality of infrastructure, and amenity facilities. Furthermore, the indicators at the city level can hardly reflect how human individuals perceive the urban environment from the micro-perspective. For example, some studies observed that people’s feelings of well-being is negatively correlated with the distance to recreation areas (such as beaches or entertainment facilities; Brereton et al., 2008), and highlighted the significant impact of urban spatial form on people’s well-being (Ma et al., 2021). However, from the perspective of individuals, the macro-level urban environmental indicators are hardly directly perceptible, resulting in the intelligibility when attempting to elucidate the impact mechanisms between such indicators and public emotions. Besides, although there are studies discussing the impact of perceptible environmental factors, such as street view and micro-level urban physical environments, on public emotions from the micro- individual perspective, most of them focused on only one or some of the environmental factors. Public emotions are comprehensively related to both objective urban environment from the macro-perspective and the individual’s perception of the urban environment from the micro-perspective. Therefore, further studies are needed to explore how urban built environment from the macro- and micro- perspective integratively impact public emotions and elucidate the complex impact mechanisms.

As to the research methods, most existing studies on public emotions employed questionnaire surveys and interviews, which can hardly support a comprehensive and real-time statistical analysis (Rahnema et al., 2019). These traditional methods have limitations for they are based on passive, static, and small-sized sample data (Duan et al., 2022). With the popularity of social media applications, people get used to sharing their daily lives and expressing their opinions on social media. In this context, location-based social network (LBSN) data emerged (such as Twitter and Weibo) and quickly presented a large amount of real-time public emotion data. These emerging social media big data make it possible to address the shortcomings of traditional methods of public opinion research (Giuffrida et al., 2020). Despite some controversies, such as privacy, representativeness, and other issues (Murthy et al., 2015), Twitter data are increasingly replacing traditional survey data as a “social sensor” to help better understand the social phenomena in the real world (Naaman et al., 2014). With the help of Natural Language Processing techniques, we can access the API port of Twitter to obtain all data with a certain keyword and understand the public opinion in an emergency. We can also count the tweets with geographical location information and filter them by latitude and longitude coordinates and time to map the spatial distribution of public emotions in a city within a certain time range (Marouane et al., 2021). Spatiotemporal analysis of public emotions by using LBSN data has become a popular research topic in urban research and has yielded notable achievements across various aspects. Examples include the relationship between the built environment and public emotions (Fan et al., 2023), the effect of public green space on public emotions (Chen S. et al., 2022), and the opinions of people on public transportation through Twitter data (Das and Zubaidi, 2023). In addition, previous studies used traditional global regression models, such as ordinary least squares (OLS) to explore the relationship between the built environment and public emotions. In recent years, with the emergence of novel modeling methods such as machine learning, recent studies have demonstrated the existence of threshold and synergistic effects between the built environment and human activities (such as travel, emotions, etc.) (Yang et al., 2020, 2024). However, it remains unclear whether there is a nonlinear relationship and a synergistic influencing mechanism between the built environment and public emotions, warranting further exploration of the intricate associations involved.

### 1.3 Research purpose

To fill the relevant research gap, this study aims to disentangle the intricate relationship between urban micro- and macro-built environmental factors and the semantic orientations of public emotions. In particular, this study explores following three aspects: (1) to study the relationship between urban built environmental factors and public emotions from both macro- and micro- perspective; (2) to examine the nonlinear relationship and threshold effect between various built environmental factors and public emotions; (3) to explore the synergistic effect between the built environmental factors. This study aims to deepen the understanding of the correlation between urban environment and the semantic orientation of public emotions and provide support for planners to improve public happiness from the perspective of urban planning.

## 2 Research framework

**Figure 1:**
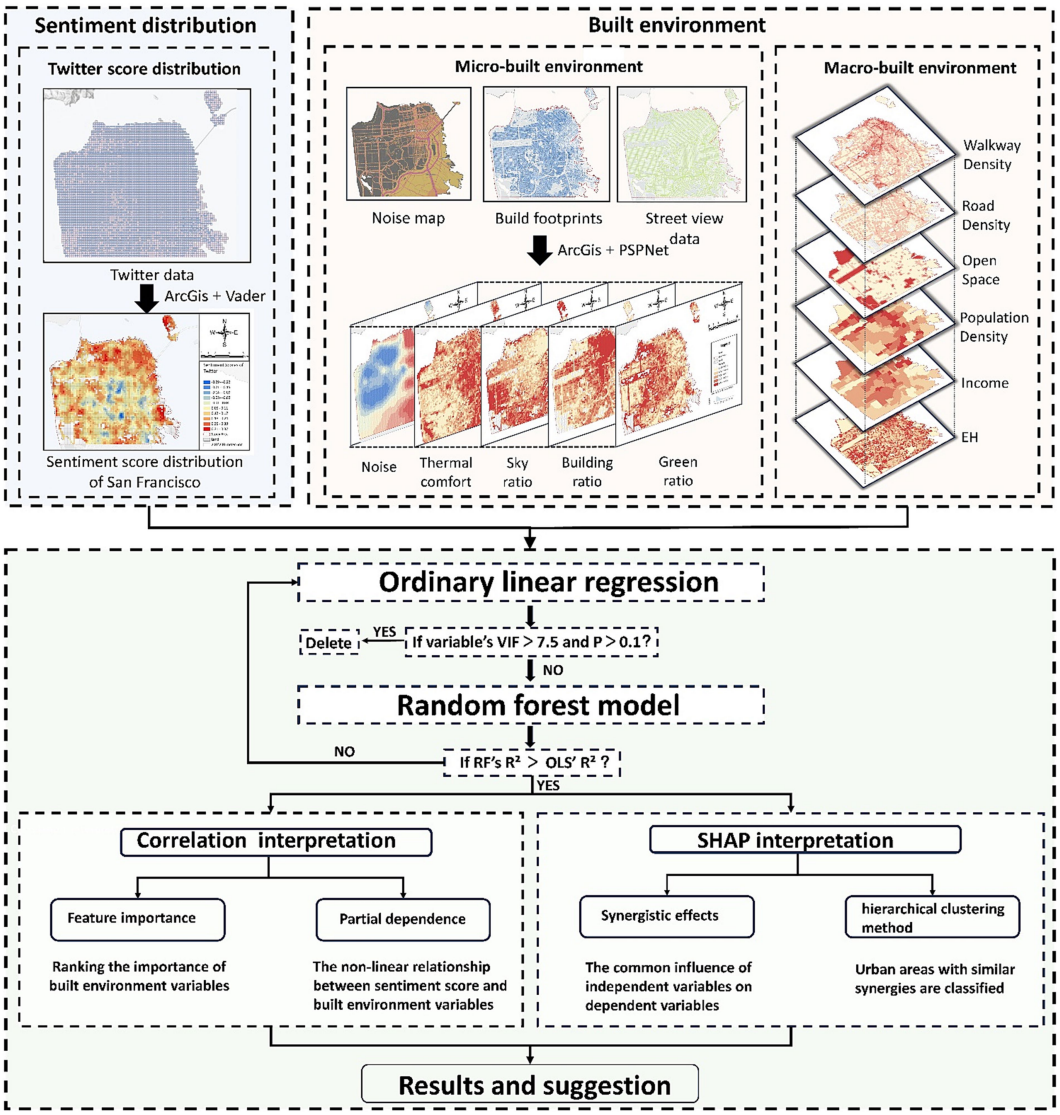
Research framework.

To achieve the research goals, the research framework is as follows:

1. First, we obtained the tweet data with geographic information in San Francisco in 2019 through the Twitter API, used the natural language processing algorithm (Vader) to quantify the polarity of the semantic orientations of tweet texts with the sentiment index, and elucidated the spatial characteristics of the public emotions according to the coordinate information of the tweet data.
2. Then, we established an urban environment indicator system combining the micro- and macro- perspective. The micro-built environmental variables included thermal comfort, street view index, and noise which were computed from diverse multi-source big data, including Landsat 8 satellite imagery, street view images, and urban noise data. The traditional macro-built environmental indicator system was built based on the classical 5D framework and was then used as control variables for testifying the impact of the micro-built environment variables.
3. Finally, based on the result of the exploration of the Ordinary Least Squares (OLS) regression model, we assumed a nonlinear relationship and synergistic effects among variables in the urban built environment and the polarities of public emotions. The Random Forest (RF) model and SHAP algorithm were employed to further reveal the complex relationship. The specific research framework is shown in Figure 1.

## 3 Data and method

### 3.1 Sentiment analysis of tweet data

**Figure 2:**
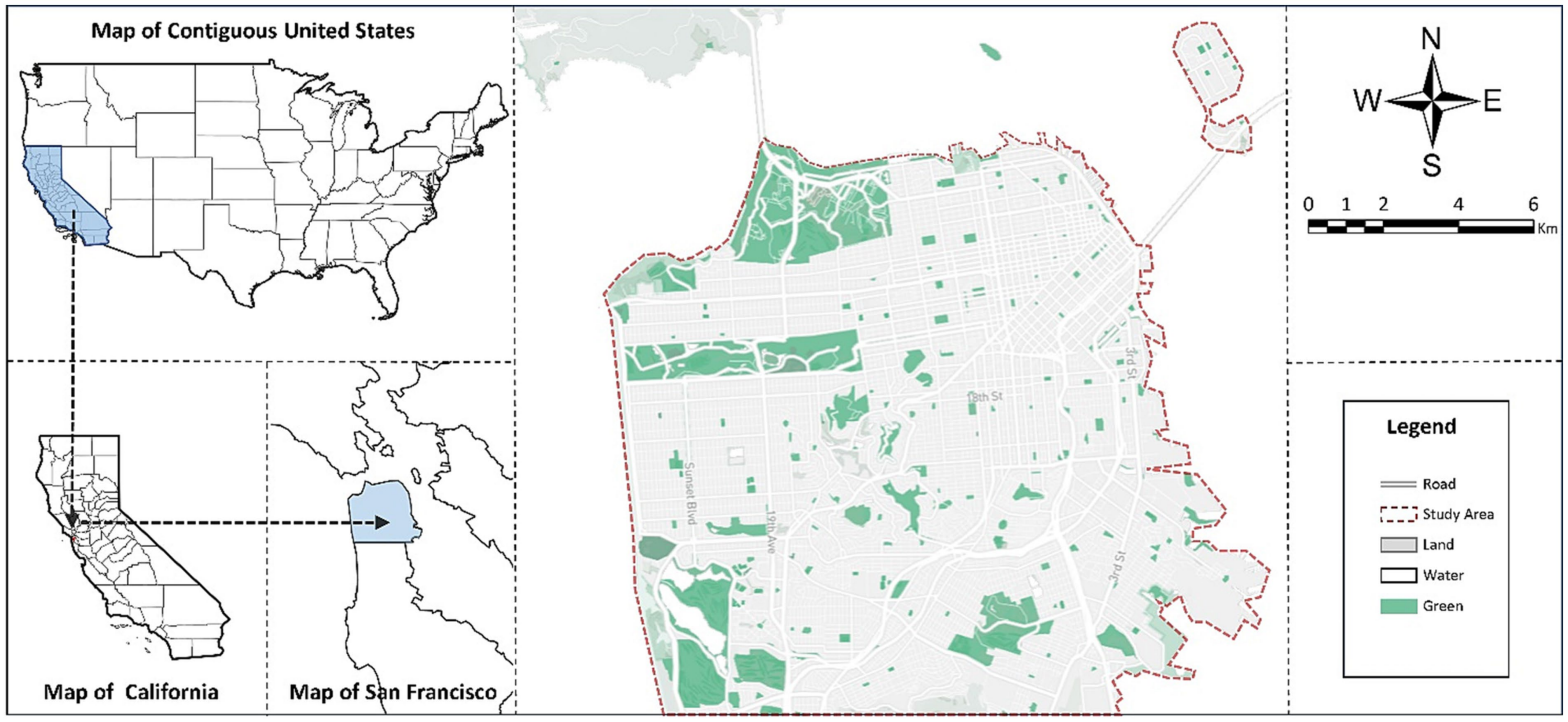
Study area.

This study was conducted in San Francisco, the fifth largest city in the United States, as shown in Figure 2. San Francisco is surrounded by sea on three sides, with an urban area of approximately 600.6 km^2^ and a permanent population of approximately 850,000. We used the Twitter Streaming API to obtain 121,270 historical tweets from San Francisco between January 1 and December 31, 2019, which was chosen to avoid the effect of the COVID-19 pandemic. All tweet data includes only the content, time, and location information of the tweets, without any involvement of users’ real information or privacy. The data were preprocessed to exclude non-text information, such as bot text, useless links, subject tags, and emoticons, to avoid interfering with data processing and model training. Finally, the cleaned data were obtained.

We used the Vader library in Python to analyze the sentiment of the cleaned tweets. Vader is a lexicon and rule-based sentiment analysis tool that is specifically attuned to sentiments or emotions expressed in social media. It employs a combination of a sentiment lexicon, grammatical rules, and syntactical heuristics to determine the semantic orientation of a given text as either positive or negative. Specifically, a sentiment is considered “positive” when the text conveys a favorable or optimistic opinion, emotion, or attitude. Examples of positive sentiments include joy, happiness, satisfaction, or admiration. On the other hand, a sentiment is considered “negative” when the text conveys an unfavorable or pessimistic opinion, emotions, or attitude, such as sadness, anger, disappointment, or frustration. Moreover, Vader not only determines the positivity and negativity of the textual sentiment, but also gives a compound sentiment score about how positive or negative a sentiment is (Hutto and Gilbert, 2015). The score ranges from −1 to 1, and the larger the absolute value of the score is, the higher the emotional intensity will be. In this study, the compound sentiment score was defined as the sentiment index measuring the intensity of positive or negative sentiment expressed in a tweet text, helping to understand the semantic orientations of overall public emotions expressed in the tweet data.

With the coordinate information of tweet data, the sentiment index of every tweet was mapped as a spatial point employing ArcGIS pro. To get a better understand of the spatial characteristics of the distribution of the sentiment index and its spatial relationship with the urban environment, this study employed a 200 m × 200 m grid as a basic statistic unit, the sentiment index and the urban environment indicators in each cell were aggregated based on the averages. In addition, the grid with a small number of samples was deleted, and the average sentiment index of each grid was obtained based on the following formula to ensure the normal distribution of data and eliminate the influence of outliers (Gai et al., 2022).


$${\hat{Y}}_{r}=\left(\frac{N}{n}\right)\left[\varphi \left(\varphi^{-1}\left(\overset{r-1}{\underset{i=1}{\sum }}\frac{n_{i}}{N}\right)\right)-\varphi \left(\varphi^{-1}\left(\overset{r}{\underset{i=1}{\sum }}\frac{n_{i}}{N}\right)\right)\right]$$

where $\varphi^{-1}$ is the inverse standard normal cumulative density function, r is the score range of positive emotion, $n_{i}$ is the number of cases in the range r , N is the total number of cases, ${\hat{Y}}_{r}$ is the normal score for range r , and φ is the standard normal density function. Higher Y values represent a stronger positive public emotion in the grid.

### 3.2 Micro-built environment variables

#### 3.2.1 Google street view images index

**Figure 3:**
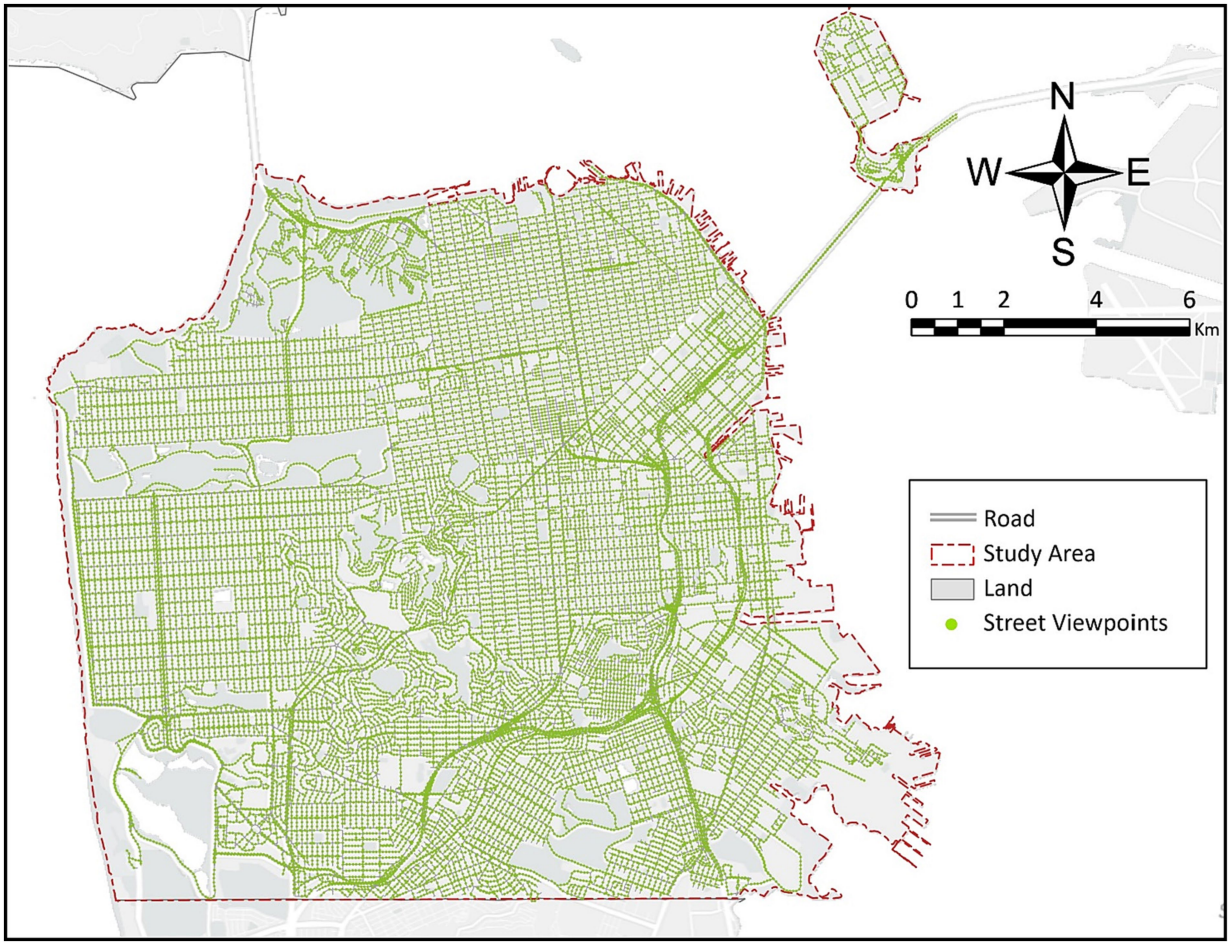
Spatial distribution of 23,171 sampling points of a street view image.

**Figure 4:**
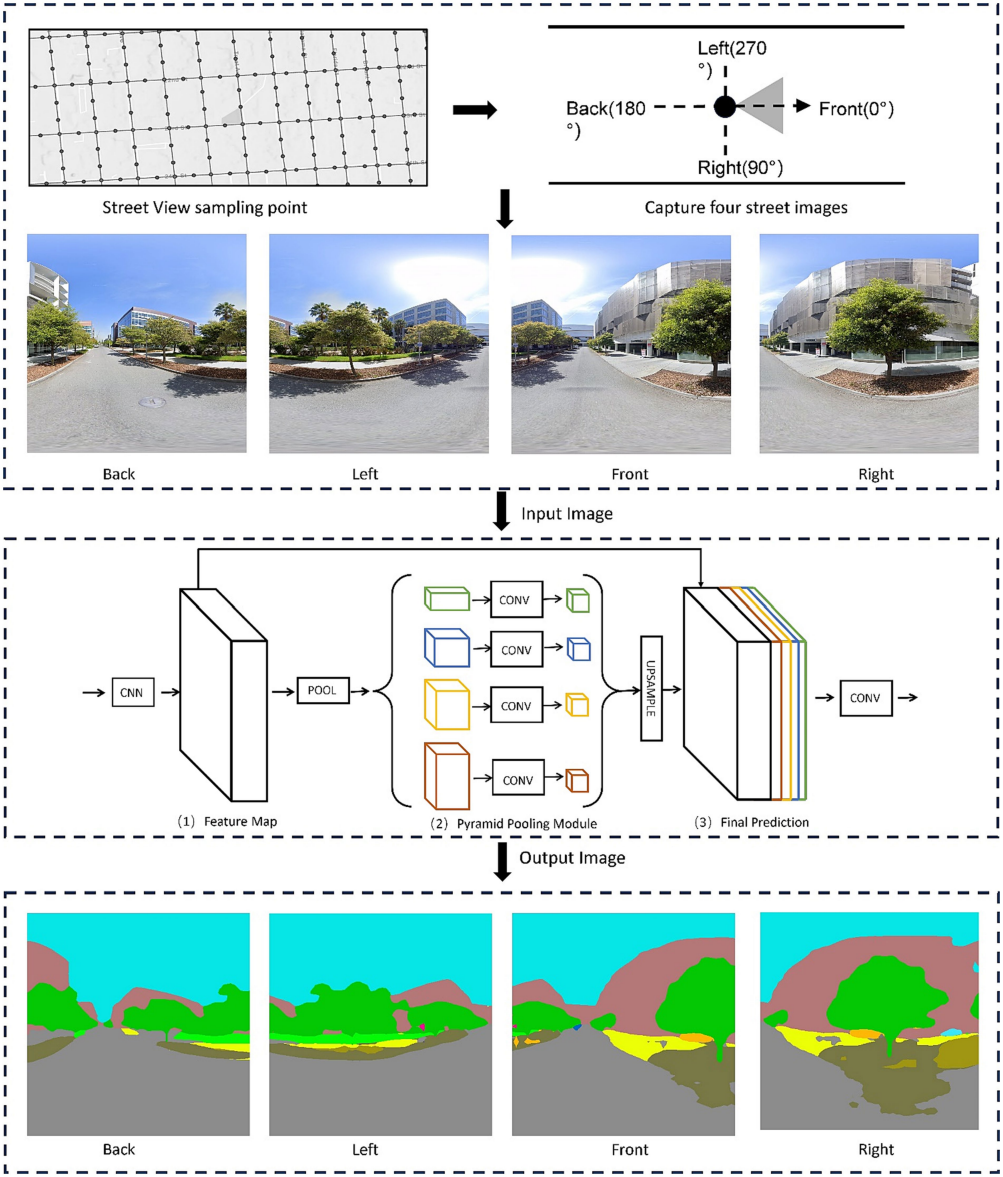
Assessing streetscape features from the PSPNet algorithm.

Real-world street view images with geographic information make it possible to quickly extract street view information from the human perspective and in large quantity (Kaneko and Yanai, 2016). Street view images have rich environmental features and can visually show the urban microenvironment. Recently, research on the quantification of the built-up environmental features of streets by combining street view images with machine learning algorithms has gradually become a major topic (Zhang et al., 2018; Chen L. et al., 2022). For example, the proportion of different environmental elements in street view images can be extracted by semantic segmentation algorithms, which can help efficiently capture the features of the micro-scale urban environments over a wider geographic area (Zhao et al., 2016). Accordingly, this study generated 23,171 sampling points within San Francisco City at 50-m intervals based on the OpenStreetMap street network and grabbed four Google Street View images from different angles at each sampling point through the Google API (Figure 3). A total of 92,684 street view images were obtained, with the angles of each direction being 0°–90°, 90°–180°, 180°–270°, and 270°–360°. The image resolution was 995 × 1,215 pixels, and the camera settings and resolution of each image remained unchanged. Thereafter, the image semantic segmentation processing was conducted using a pretrained Pyramid Scene Parsing Network (PSPNet) model based on the Ade20k dataset (Qiu et al., 2022; Figure 4). Every pixel in an image was labeled with a class number representing its visual category, such as tree, building, and road. For the study purpose, the segmentation results were filtered and reclassified, and the proportion of pixels that could be classified as sky, building and greenery were calculated, respectively. Specifically, the building includes pixels that were labeled as building, wall, house, skyscraper, shanty, tower and shelter, the sky refers to pixels that were labeled as sky, and the greenery refers to pixels that were labeled as tree, grass, plant and flowers. For each sampling point, the sky ratio, the building ratio and the green ratio were calculated based on the average proportion of the corresponding category of pixels in four images of different orientations. The calculation formula for the street view index system is as follows (Luo et al., 2023):


$$Viewindex=\frac{\sum_{i=1}^{4}characterPixels}{\sum_{i=1}^{4}TotalPixels}$$

where the view index is the proportion of environment character pixels in four images, and i is the number of images.

Finally, the three street view indicators, namely the sky ratio, the building ratio, and the green ratio, were aggregated into each of 200 m × 200 m gird cells based on the average, as descriptors of the street-level visual environment from human perspective.

#### 3.2.2 Noise data

**Figure 5:**
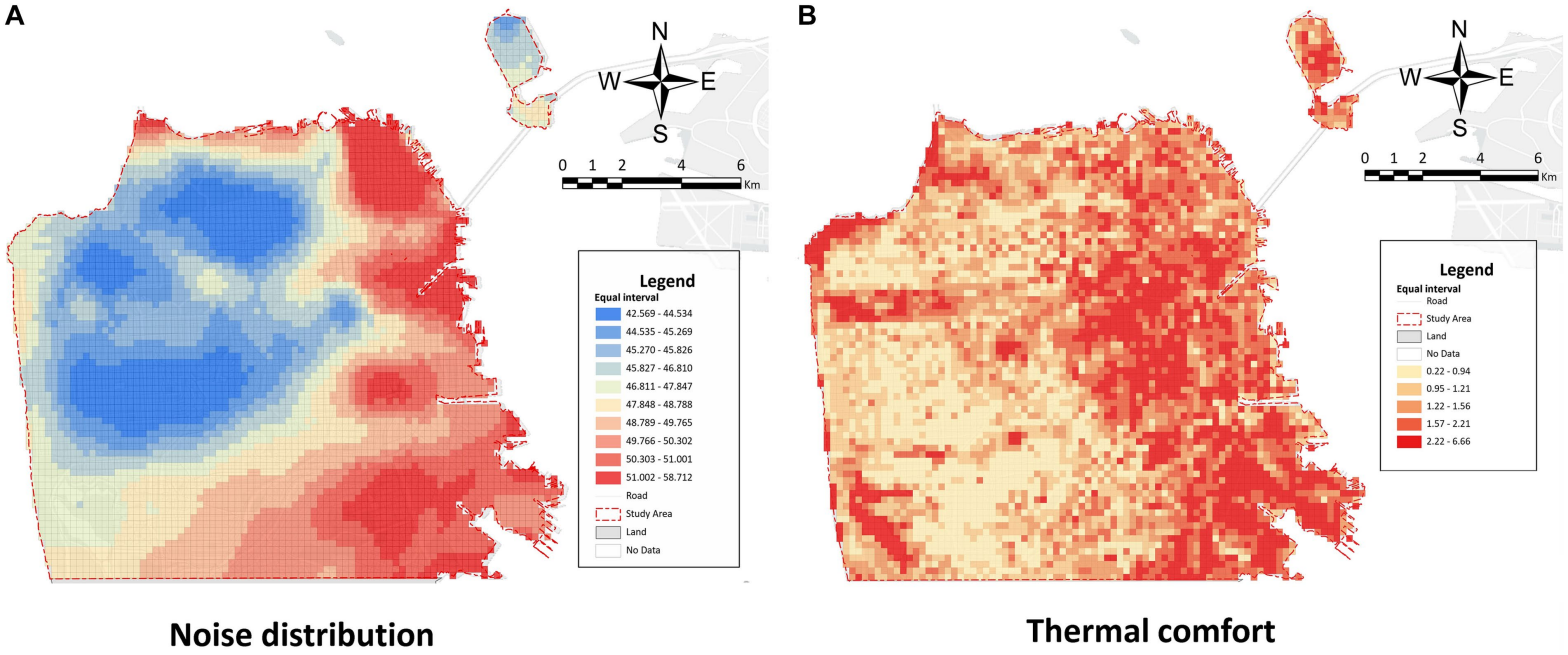
Spatial distribution of physical environmental variables in San Francisco. **(A)** the spatial distribution of noise; **(B)** the spatial distribution of thermal comfort.

A plethora of evidence has demonstrated that long-term exposure to urban noise has a negative influence on people’s mental health, resulting in feelings of irritability, anger, and even depression (Huang D. et al., 2023). However, the high cost of land determines that the cities will keep developing toward compactness and density (Haaland and Van Den Bosch, 2015). Consequently, cities are likely to become more densely populated over time, the urban noise pollution problem will become severe correspondingly, which can significantly inhibit the daily emotion of urban residents. On this basis, we obtained the OpenStreetMap urban noise data from the website “noise-map.com” to measure the urban acoustic environment in San Francisco. Noise-map.com is a visual website of urban noise data. This website evaluates and visualizes the noise pollution generated in urban environments and from aircraft and other transport means based on real sensor information and transport information. We used the noise data from the noise-map to examine the spatial characteristics of noise pollution in San Francisco. The noise decibel data were resampled into the 200 m × 200 m grid based on the mean value (Figure 5).

#### 3.2.3 Thermal comfort data

The heat island effect and extreme high temperature are becoming increasingly severe, bringing great challenges to environmental sustainability (Luo et al., 2023). The temperature in the urban center area (especially in summer) is typically higher than the surrounding areas, bringing numerous negative effects to human outdoor activities, such as thermal radiation diseases and emotional irritability (Chiang et al., 2023). At present, a commonly used method of evaluating the urban thermal comfort environment is to calculate the urban surface temperature based on the satellite remote sensing images, such as Landsat 8 satellite images and MODIS satellite images. However, there is always a tradeoff between the spatial and temporal resolution of satellite images. The spatial resolution of the Landsat 8 satellite reaches 30 m, but it revisits the same observation area and takes an image every 16 days. On the contrary, the MODIS satellites have a temporal resolution of 1–2 days, but their spatial resolution is 1 km. Considering that the city scale (land area) of San Francisco is approximately 120 km^2^, the spatial resolution of the MODIS satellite was considered to be insufficient to reflect the thermal characteristics of the microenvironment from a human perspective. Accordingly, we opted for the Landsat 8 satellite. This study obtained Landsat 8 images of San Francisco in 2019, eliminating cloudy and night images. The satellite images were divided into four seasons (spring: March 21st–June 21st, summer: June 22nd–September 22nd, autumn: September 23rd–December 21st, and winter: December 22nd–March 20th) and the average surface temperatures of each season were calculated and resampled to the 200 m × 200 m grid. Since the comfortable temperature for the human body varies in each season, to make the indicator better interpretable, we subtracted the threshold of comfortable temperature from the surface temperature in each grid cell to get the disparity from the comfortable temperature range. Finally, this study measured the thermal comfort in each grid cell by calculating the average absolute difference with the most comfortable temperature in the four seasons. The smaller thermal comfort index, the fewer disparities with the most comfortable temperature, the more comfortable the thermal environment in the grid cell. The formula is as follows:


$$Atc\;index=\frac{\sum_{i=1}^{4}\left|a_{i}-b_{i}\right|}{4}$$

where the *Atc* index is the average thermal comfort index, a is land surface temperature, and b is comfortable temperature. According to previous research conclusions, the thermal comfort zone in the moderate climate zone of the United States in summer, namely, the dry bulb temperature, is between 22°C and 28°C. Meanwhile, in winter, the dry bulb temperature under the same conditions is between 20°C and 25°C (Potchter et al., 2018). Therefore, we take the above range as the most comfortable temperature range for winter and summer and 20°C–28°C for the two transitional seasons of spring and autumn. Finally, the spatial distribution of thermal comfort for each grid is shown in Figure 5.

### 3.3 Macro-built environmental variables

In urban environmental research, the 5D (density, diversity, design, destination accessibility, and distance to transit) framework is widely used for the systematic classification of macro built environment variables (Guzman et al., 2020; Yu et al., 2022). In addition, this study combined socio-economic aspects with the classical 5D framework and preliminarily constructed a set of macro-built environment indicators as control variables to explore their correlation with public emotions. Specifically, density includes population density and building density. Diversity includes the land use diversity, the walkway density, and the green land ratio. The design includes the floor area ratio and the open space ratio. Accessibility includes the road density, the distance to the city center, and the distance to sea.

The original data used to construct the macro- built environment indicators were all from the official public data of the US government.^1^ The original data were aggregated into the 200 m × 200 m grid by mean values. For data with different scales, such as the census tract population, we conducted a resampling process using a weighted average based on the proportion of the overlapping areas with each grid cell. In addition, the land use diversity was based on the Shannon Diversity Index $E_{H}$ , measuring the degree of diversification of land use types, and its calculation formula is as follows (Blair and Launer, 1997):


$$E_{H}=\frac{-\sum \left(P_{i}\right)\ln P_{i}}{\ln\left(S\right)}$$

where $E_{H}$ is the diversity index, $P_{i}$ is the ratio of the i type of land to the total area, and S is the total number of unique land use types. The greater the land use diversity index, the higher degree the land uses are mixed in the study unit.

**Figure 6:**
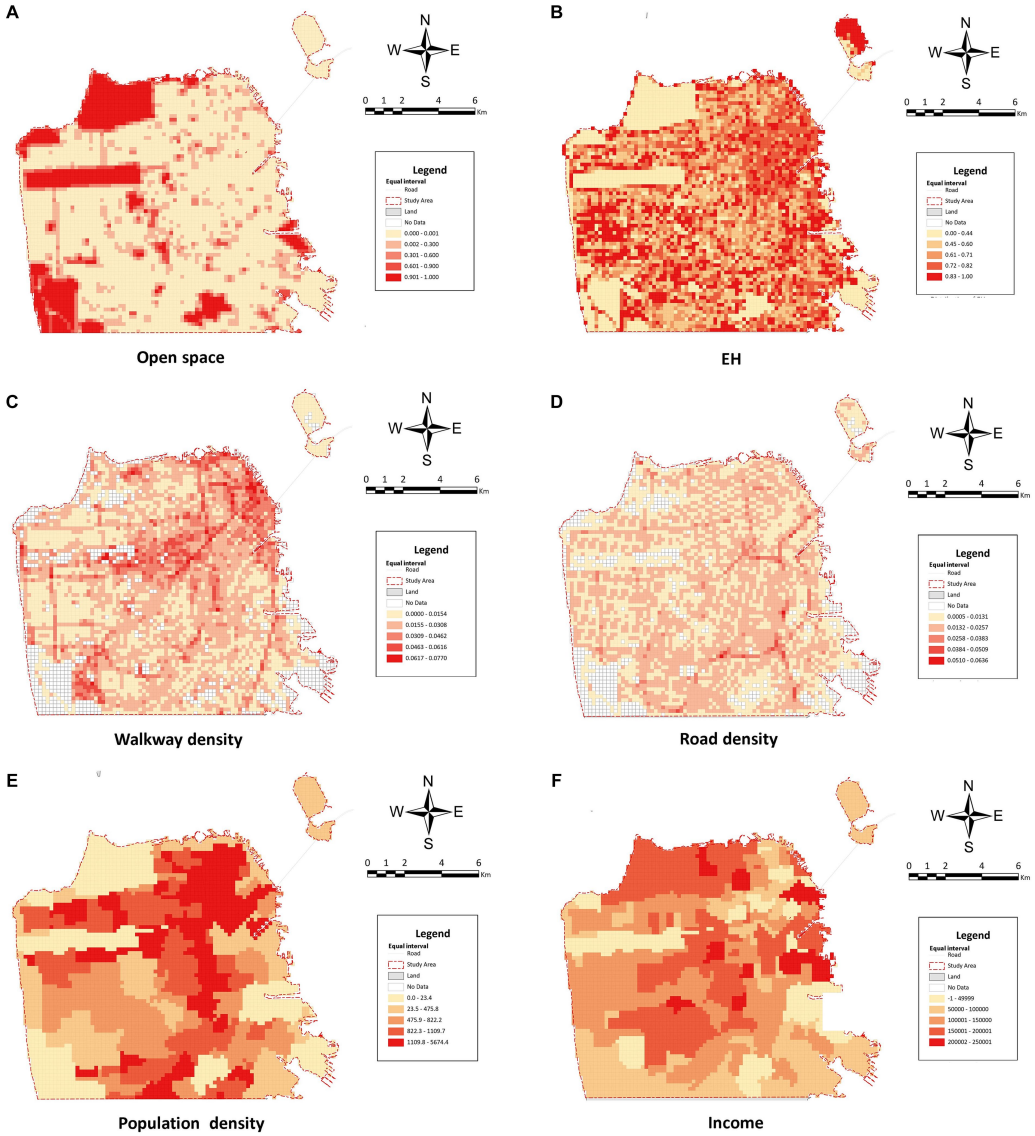
Spatial distribution of the macro-built environmental variables. **(A)** Open space; **(B)** EH the Shannon Diversity Index of land use; **(C)** Walkway density; **(D)** Road density; **(E)** Population density; **(F)** Income.

Before model building, we examined the collinearity between variables based on a correlation analysis. In particular, San Francisco is surrounded by the sea on three sides, and the downtown area is located to the northeast of the city center. The distance to the downtown area and the distance to sea are exactly opposite variables, with a strong negative correlation. Considering that the distance to the coastline also represents the distance to the beach and the relevant recreation places, we excluded the variable of the distance to the downtown area. In addition, a strong correlation existed between the building density, floor area ratio, and the building ratio index in the microenvironmental variables. Furthermore, the green land ratio and the Streetview green ratio index were also highly correlated. Consequently, the variables of the building density, the floor area ratio, and the green land ratio were excluded, the final indicator system for the micro- and macro- built environment and the descriptive statistics are shown in Table 1. The spatial distribution of macro-built environmental variable data is shown in Figure 6.

**Table 1:** Descriptive statistics of the variables.

Variables	Obs.	Mean	Std.	Min	Max
Dependent variables					
Sentiment score	4,231	0.139	0.0909	−0.286	0.369
Independent variables					
Macro-built environmental variables					
Distance to sea	4,231	2597.996	1783.724	3.138	6614.741
Income	4,231	121107.63	58442.361	0	250,001
Population density	4,231	0.00779	0.00629	6.74E-06	0.0567
Road density	4,231	0.0137	0.00629	0.000474	0.0635
EH	4,231	0.948	0.442	0	2.034
Open space	4,231	0.133	0.291	0	1
Walkway density	4,231	0.0222	0.0124	0	0.0769
Micro-built environmental variables					
Sky ratio	4,231	0.372	0.0621	0.0574	0.498
Green ratio	4,231	0.0745	0.0677	0	0.493
Building ratio	4,231	0.0939	0.0591	0	0.394
Noise	4,231	47.740	2.631	42.568	58.711
Thermal comfort	4,231	1.534	0.739	0.257	5.985

### 3.4 Method

After the data preprocessing, we conducted an exploration regression on all variables by using the OLS model and further tested the multicollinearity problem according to the correlation coefficient (P) and the variance inflation factor (VIF) of each variable (Park et al., 2018). If the *p* value of a variable is greater than 0.1, then it is insignificant. If the VIF is greater than 7.5, then it is multicollinear. The variables that suggested multicollinearity (VIF > 7.5) and not statistically significant (*p* > 0.1) were eliminated for further analysis. Then, this study developed two OLS models: one containing all variables and another that includes only the micro-built environmental variables. The two OLS models were compared to examine the impact of micro-built environmental variables on public emotions.

We used the Random Forest (RF) model to explore the complex relationship between the built environmental variables and public emotions. RF is one of the most powerful and popular machine learning algorithms that can process high-dimensional data (Biau, 2012). This algorithm is an extension of Bagging (a parallel ensemble learning method) and uses a classification regression tree algorithm (CART) as the basic learner to form the entire tree model. RF is highly robust because it can model different data types and is insensitive to multiple collinearities, missing values, outliers, and irrelevant variables (Breiman, 2001). First, the RF algorithm extracts a certain proportion of samples from the original dataset to form the sample training and test sets. Second, when building the decision tree model, the RF algorithm generates a set of decision trees. Each set of decision trees is trained on a bootstrap sample from the original dataset, and the optimal node splitting variable is selected from a random subset of all independent variables. Finally, the RF model generates the final prediction by averaging all predictions of the basic CART, whose calculation principle is shown in the following formula:


$${\hat{f}}_{s}\left(x_{s}\right)=E_{x_{c}}\left[f\left(x_{s},X_{C}\right)\right]=\int f\left(x_{s},x_{C}\right)p\left(x_{C}\right)dx_{C}$$

$${\hat{f}}_{s}\left(x_{s}\right)\approx \frac{1}{M}\overset{M}{\underset{m=1}{\sum }}f\left(x_{s},x_{C}^{\left(m\right)}\right)$$

where f defines the function of the machine learning model, $x_{s}$ denotes the one or two features of interest, $x_{c}$ is the set of other features, $X_{C}$ is used in the model, ${\hat{f}}_{s}\left(x_{s}\right)$ is the partial dependence function for regression at point $x_{s}$ , $f\left(x_{s},x_{C}^{\left(m\right)}\right)$ is the model prediction for a specific mth sample whose feature values are determined by $x_{s}$ and $x_{C}$ , and M is the number of samples.

RF models have a host of strengths. First, RF models do not prescribe some kind of correlation between the independent and dependent variables. Accordingly, this model can capture the potential complex nonlinear relationship between the two, which is impossible for traditional linear regression models to do. Second, the advantages of RF compared with other tree models (such as GBDT) are its insensitivity to outliers, ability to handle high-dimensional data, being less prone to overfitting, and being highly adaptable to the dataset: it can handle discrete and continuous data, and the dataset does not need to be normalized. In summary, we employed the RF model to further explore the complex relationship between the built environment and public emotions. Finally, two important data results are outputted: the Shapley value and the partial dependence graph (PDP). Shapley Additive Explanations (SHAP) is a representative interpretable model (Lundberg and Lee, 2017). SHAP can more intuitively represent the local positive or negative effect of all samples of a variable on the model compared with the previous variable importance ranking model and further decompose it into the interaction between the main local effect of the variable and other variables. Meanwhile, PDP can describe the degree of correlation change of variables in different value ranges without assuming a linear correlation (Yang et al., 2023), which helps in improving the interpretability of the model.

## 4 Results

### 4.1 Distribution of sentiment index

**Figure 7:**
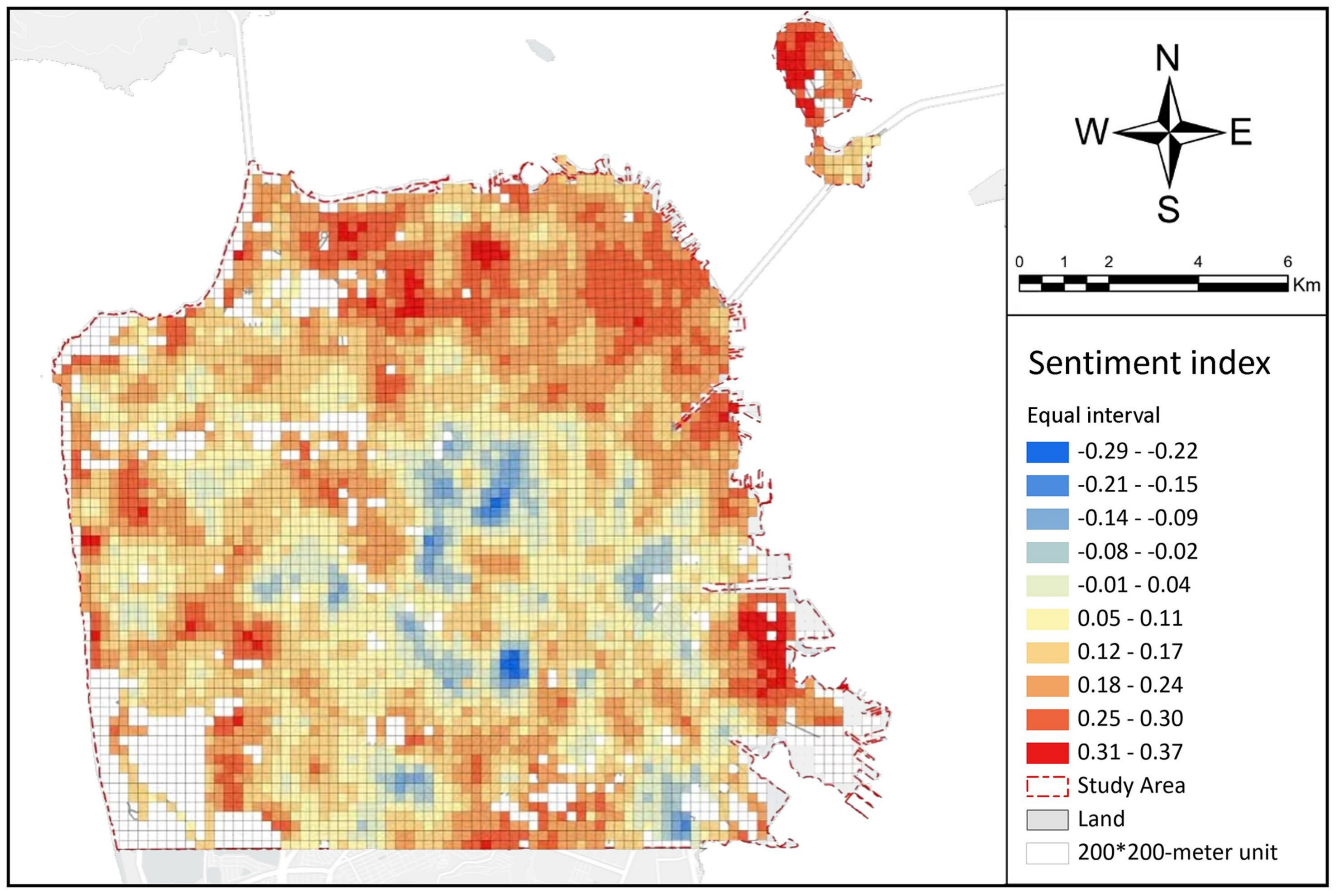
Spatial distribution of sentiment index.

Figure 7 displays the distribution of the public sentiment index in San Francisco. As the figure shown, the sentiment index ranges from −0.29 to 0.37. Considering the threshold of the sentiment index between −1 and 1, the public sentiment index over all grid cells suggests limited variations. However, this is because we took the mean value of the sentiment scores in each grid cell, which averaged out the extreme values. Nevertheless, we still observed significant spatial heterogeneity in its distribution across the grid cells. For better visualization and easier understanding, the sentiment index of the grids is divided into 10 classes with equal interval, a deeper blue color in a grid cell suggests people in the corresponding area tend to have more negative emotions, while a deeper red color in a grid cell indicates people in the corresponding area generate more positive emotions. Notably, the average sentiment index across the grids is 0.139, with a standard deviation of 0.09, indicating an overall positive emotions distribution in San Francisco (Table 1). Figure 7 implies potential spatial correlations between the urban environmental factors and the public sentiment index. For example, the public sentiment index in the northern and northwestern areas of the city, including Marina, North Beach, and Civic Center, are higher than those in the central and southern regions. Additionally, areas near the coastline have a significantly higher sentiment index compared with the central areas farther from the coastline. Moreover, places abundant in vegetation resources exhibit a notably higher sentiment index than areas with limited vegetation resources. These findings suggest that people in areas with specific environmental conditions are more likely to have positive emotions than other areas. However, these findings might not be universally applicable because each city possesses its own distinct social, geographical, and economic attributes. For instance, a study conducted in Bhopal, India revealed that the distance to open spaces and the proximity to slums are the primary determinants of public emotion distribution based on sentiment analysis of Twitter data in that city (Khare and Chatterjee, 2023). Therefore, further studies are needed to elucidate the complex relationship between the environmental factors and the public emotions.

### 4.2 Results of the OLS and RF models

Table 2 compares the results of two OLS regression models, namely, the model of all built environmental variables and the model of the micro-built environmental variables. The explanatory power of all built environmental variables for the public sentiment index is 21.1%, with each variable having a VIF below 7.5, indicating no multiple collinearity issues between variables. Three micro-built and six macro-built environmental variables show significant correlations with the public sentiment index (*p* < 0.1). From the micro-built environment aspects, the green ratio index (Coef = 0.0592, *p* < 0.05) and building ratio index (Coef = 0.1298, *p* < 0.01) demonstrate a positive correlation with the sentiment index. Thermal comfort (Coef = −0.1185, *p* < 0.05) negatively affects the sentiment index, while the sky ratio and the noise exhibit no significant correlation with the sentiment index. In terms of the macro-built environment, the sentiment index is significantly positively correlated with the open space, the land use diversity, and the walkway density. Meanwhile, there is a significant negative correlation between the sentiment index and both road density and distance to the sea. Besides, this study also observed a negative correlation between income and the sentiment index, which differs from previous research (Ngamaba, 2016) and warrants further discussion.

**Table 2:** Results of the OLS models.

	BE OLS model				Micro-BE OLS model			
Variables	Coef.	St. Er.	p-Value	VIF	Coef.	St. Er.	p-Value	VIF
Micro-BE variables								
Noise	0.0028	0.009	0.756	1.376	0.0501	0.009	0.000	1.222
Thermal comfort	−0.119^***^	0.0011	0.000	1.260	−0.0487^***^	0.011	0.000	1.176
Sky ratio	0.0030	0.024	0.898	7.264	−0.0212	0.025	0.402	7.015
Green ratio	0.0428^*^	0.025	0.086	7.327	−0.0385	0.026	0.145	7.240
Building ratio	0.1108^***^	0.023	0.000	6.92	0.0993^***^	0.024	0.000	6.896
Macro-BE variables								
Distance to sea	−0.1270^***^	0.005	0.000	1.315				
Income	−0.0263^***^	0.006	0.000	1.172				
Population density	0.0157	0.014	0.274	1.355				
Road density	−0.1369^***^	0.014	0.000	1.355				
EH	0.0141^**^	0.007	0.032					
Open space	0.0401^***^	0.007	0.000	2.398				
Walkway density	0.0289^***^	0.009	0.001	1.363				
R-squared	0.211				0.071			
Log-likelihood	4646.8				4295.2			
AIC	−9,268				−8,576			

^***^p < 0.01; ^**^p < 0.05; ^*^p < 0.1.

After the exploration regression of OLS models, we import the sample data into the RF model for cross-validation and hyperparameter tuning to optimize the model’s performance. As a result, this study adopted the following hyperparameters to achieve a sound result without overfitting: 80% of the sample was allocated to the training set, while the remaining 20% was the test set. The number of learners (n_estimators) was 100, and the max_features was set to 3. Table 3 presents the model performance metrics, including MAE of 0.039, MSE of 0.0027, MAPE of 1.092, and an R^2^ value of 0.6819. These results indicate that the model exhibits good accuracy and possesses predictive capabilities. Moreover, compared with the OLS models, the RF model significantly fits better with a higher R^2^, implying that there exists a complex relationship between the built environment variables and the sentiment index that OLS models cannot explain. Therefore, the SHAP and PDP analyses are conducted based on the RF model to further interpret the complex relationship between the built environment variables and the sentiment index.

**Table 3:** Performance of the RF models.

	MAE	MSE	MAPE	R-squared	Train
RF	0.039	0.0027	1.092	0.68	0.8

### 4.3 Relative importance of the sentiment score

**Figure 8:**
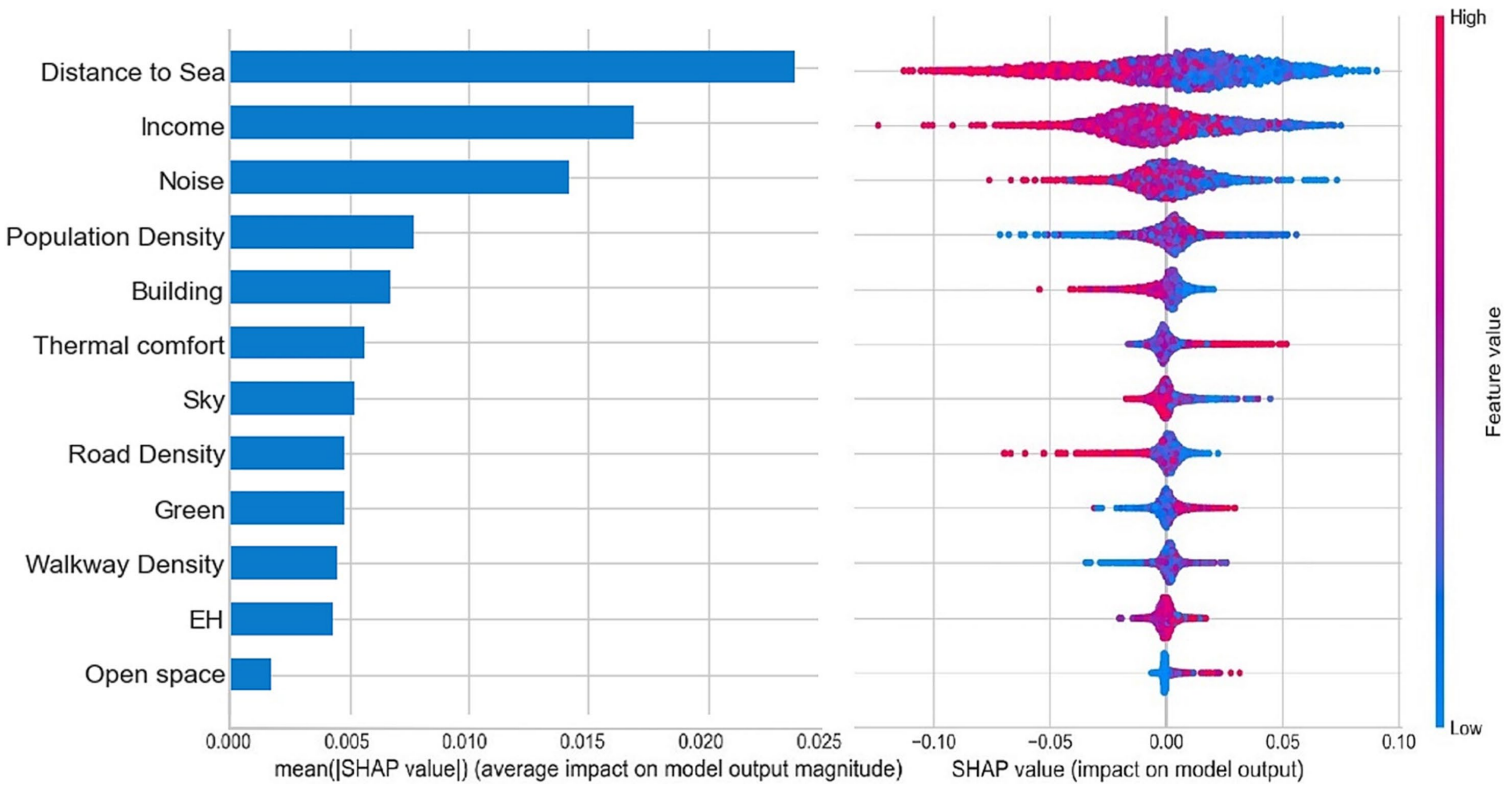
Relative importance of independent variables and a summary of local explanations.

Figure 8 illustrates the relative importance and the ranking of the built environmental factors when predicting the sentiment index in the RF model, with all independent variables contributing to a total importance of 100%. On the left part of Figure 8, the variables are arranged in descending order based on the average value of the global feature importance, calculated by the weighted average of the absolute Shapley value of each sample. This average indicates the variable’s overall contribution to the model. The specific contribution values for each variable are listed in Table 4. The macro-built environmental factors account for 61.8% of the total, while the micro-built environmental factors contribute 38.2%. This significant difference in influence between the macro and micro factors highlights the essential role of both in affecting public emotions.

**Table 4:** Relative importance of independent variables.

Categories	Variable	Sentiment score		
		Rank	Relative importance (%)	Total
Macro-BE variables	Distance to sea	1	23.6	
	Income	2	16.9	
	Population density	4	7.7	
	Road density	8	4.8	
	Walkway density	10	4.5	
	EH	11	4.3	
				61.8
Micro-BE variables	Noise	3	14.2	
	Building ratio	5	6.7	
	Thermal comfort	6	5.6	
	Sky ratio	7	5.2	
	Green ratio	9	4.8	
	Open space	12	1.7	
				38.2

Among the macro-built environmental factors, the distance to sea is the most important variable, with an effect of 23.6% on predicting the sentiment index. This finding is consistent with the conclusions of previous studies (Brereton et al., 2008; Vlker and Kistemann, 2013), highlighting that people’s feelings of well-being is significantly related to the distance to beaches and other recreation places. San Francisco is surrounded by the sea on three sides and has rich natural resources, such as the ocean and green land. In comparison with other cities, people living in San Francisco are more likely to be affected by the richness of the natural environment in their living areas, which can also be confirmed from the spatial distribution of the sentiment index in Section 4.1. Additionally, income (16.9%), population density (7.7%), road density (4.8%), and land use diversity (4.3%) emerge as subsequent important macro-built environmental variables, with open space (1.7%) having a relatively lower importance. These conclusions align with expectations and existing research.

Streetview indicators suggest the most significant (16.7% overall) impact among the micro-built environmental variables. In the three streetscape indicators affecting the sentiment index, the building ratio index holds the highest importance (6.7%), followed by the sky ratio index (5.2%) and the green ratio index (4.8%). This finding underscores the significance of visual perception when studying and evaluating the micro-built environment. Noise is the second-most important factor, affecting the sentiment index by 14.2%. This notion supports the findings of Roe et al. (2020), indicating that the noise in the micro-built environment significantly influences human emotion and even mental health. Although thermal comfort is equally important (5.6%), it is lower than our expectations and the reports in relevant existing research. This discrepancy may be attributed to San Francisco’s pleasant climate, characterized by a minimal temperature variation throughout the year, resulting in a reduced impact of the temperature difference on the sentiment index compared with cities with significant temperature fluctuations.

On the right part of Figure 8, each dot represents a sample. The vertical axis represents different built environmental variables, while the x-axis shows the sample’s Shapley value of each variable (i.e., local effect). The color indicates the feature value’s size (red for high value, blue for low value, and purple for median value). The direction on both sides of the axis indicates the positive or negative effect. This summary chart partially reveals the strength, distribution, and direction of the impact. For instance, the red line on the left side of the distance to sea variable is longer than the blue line on the right side, indicating that high-value samples significantly influence people’s emotions more than low-value samples. Moreover, the Shapley value of high-value samples far from the coastline is mostly negative, while that of low-value samples is predominantly positive. This notion indicates that the high-value samples inhibit positive emotions, while the low-value ones promote positive emotions. In summary, a significant negative correlation exists between the distance to sea and the public sentiment index. Although this chart offers valuable information, it cannot precisely quantify the effect of different value ranges on the sentiment index. Therefore, this study employed the local dependence chart to elucidate the influence of the local effect on sentiment index changes.

### 4.4 PDPs between the built environmental variables and the sentiment index

**Figure 9:**
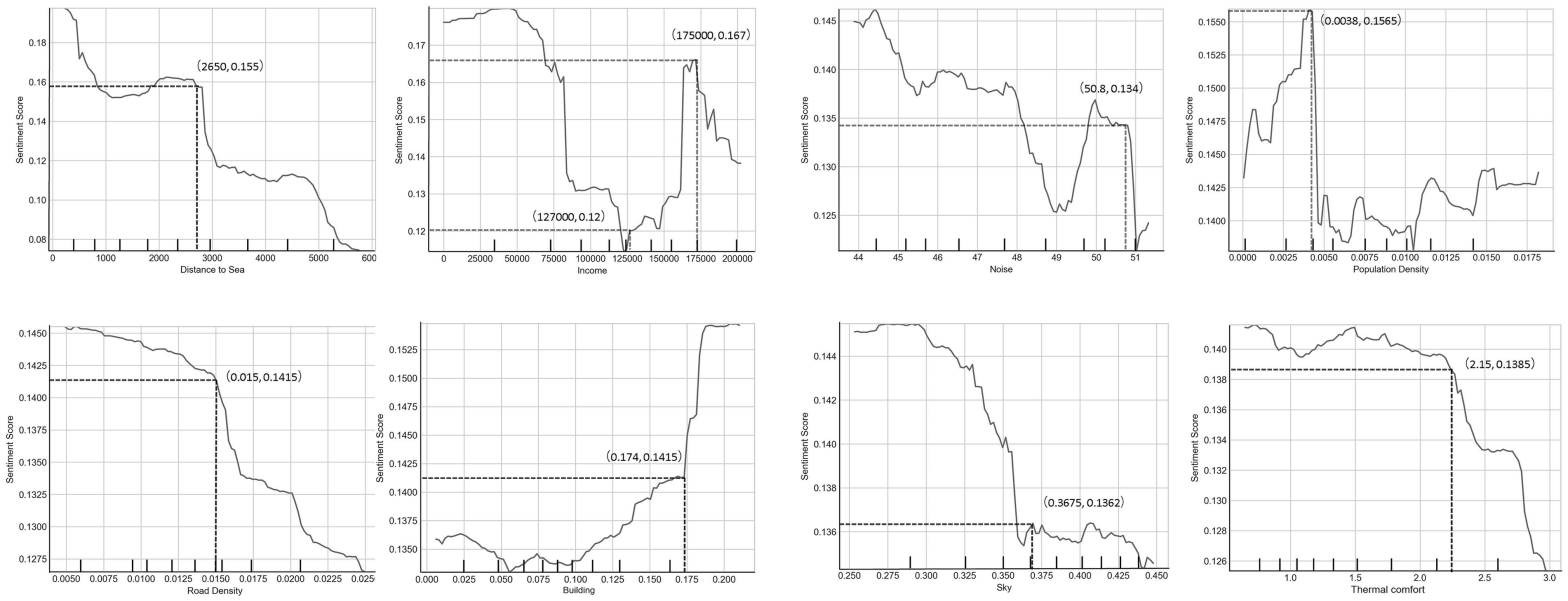
PDPs of the built environmental variables.

We calculated the interaction of each factor on the independent variable and visualized the complex nonlinear relationship between the two using PDPs, as shown in Figure 9. Each PDP corresponds to an independent variable of the built environment, demonstrating how the variable influences the sentiment index at different values. Most variables exhibit a nonlinear relationship with the sentiment index, with evident threshold effects, demonstrating that the correlation slope differs in various ranges. Among the macro-built environmental variables, the distance to sea and the road density both show a downward trend, indicating negative correlations with the sentiment index. When the distance to sea is greater than 2.8 km, the interaction effect becomes more prominent, suggesting a marginal effect of the distance to sea, namely, the shortening of the distance to sea will no longer bring additional impact on promoting individuals’ positive emotions when one already lives in proximity to a certain range (2.8 km) of coastlines. In the proportion of road density exceeding 0.015, the slope significantly increases, and the two variables are close to a negative linear relationship. This indicates that people may have a threshold to the acceptance of road density, when the road density surpasses the threshold, the corresponding transportation environment will have a more pronounced influence on emotions. The negative impact between the income and the sentiment index changes turns positive at $125,000. Below this threshold, the income and the sentiment index exhibit a negative correlation. This finding is inconsistent with common sense and previous research (Ball and Chernova, 2008). We found that the median household income in San Francisco is approximately $121,107, which is close to this threshold. Therefore, this inconsistency may be related to the corresponding nature of work at different income levels. To be specific, residents with an income below the average level are more likely to engage in more strenuous manual labor, and the increase in income usually means a longer work time or a larger workload, which causes more negative emotions. However, the incomes of the high-income individuals are not necessarily correlated with the work hours or workload, their income growth is more likely to be driven by favorable external factors, such as an upward trend in the stock market. Hence, the relationship between income and the sentiment index is complex, the income heterogeneity and inequality requires further discussion (Schurer and Yong, 2016). Besides, the appropriate high population density can enhance street vitality thus promoting the generation of positive emotions. The promotion effect sharply diminishes after the density exceeds 0.0035 person/m^2^, suggesting that overcrowding resulting from increased population density inhibits further improvement in positive emotions. Finally, the land use diversity is also positively correlated with the sentiment index. When the land use diversity index exceeds 0.38, mixed land use can significantly trigger more socioeconomic activities, promoting the generation of positive emotions.

In the micro-built environmental variables, the noise, the thermal comfort, and the sky ratio all show a trend of downward impact on the sentiment index, indicating that they are significantly negatively correlated with the sentiment index. The results about the noise and the thermal comfort are consistent with the study’s expectations. Specifically, the increase of the noise index (i.e., the noise decibel value) and the thermal comfort index (i.e., the difference from the comfortable temperature) would have a significant negative effect on the sentiment index. Unlike social, cultural, urban, and other factors, this negative effect is directly caused by physiological factors, but it is often disregarded in previous studies. The increase in the sky ratio index means a decrease in the proportion of other cityscape elements, such as buildings and green space, in city street views, the visual experience of desolation and emptiness can bring up negative emotions. On the other hand, the increase of greenery visibility in the environment can make people happy, which has been unanimously recognized by scholars (He et al., 2022).

### 4.5 Interaction effects among built environmental variables

**Figure 10:**
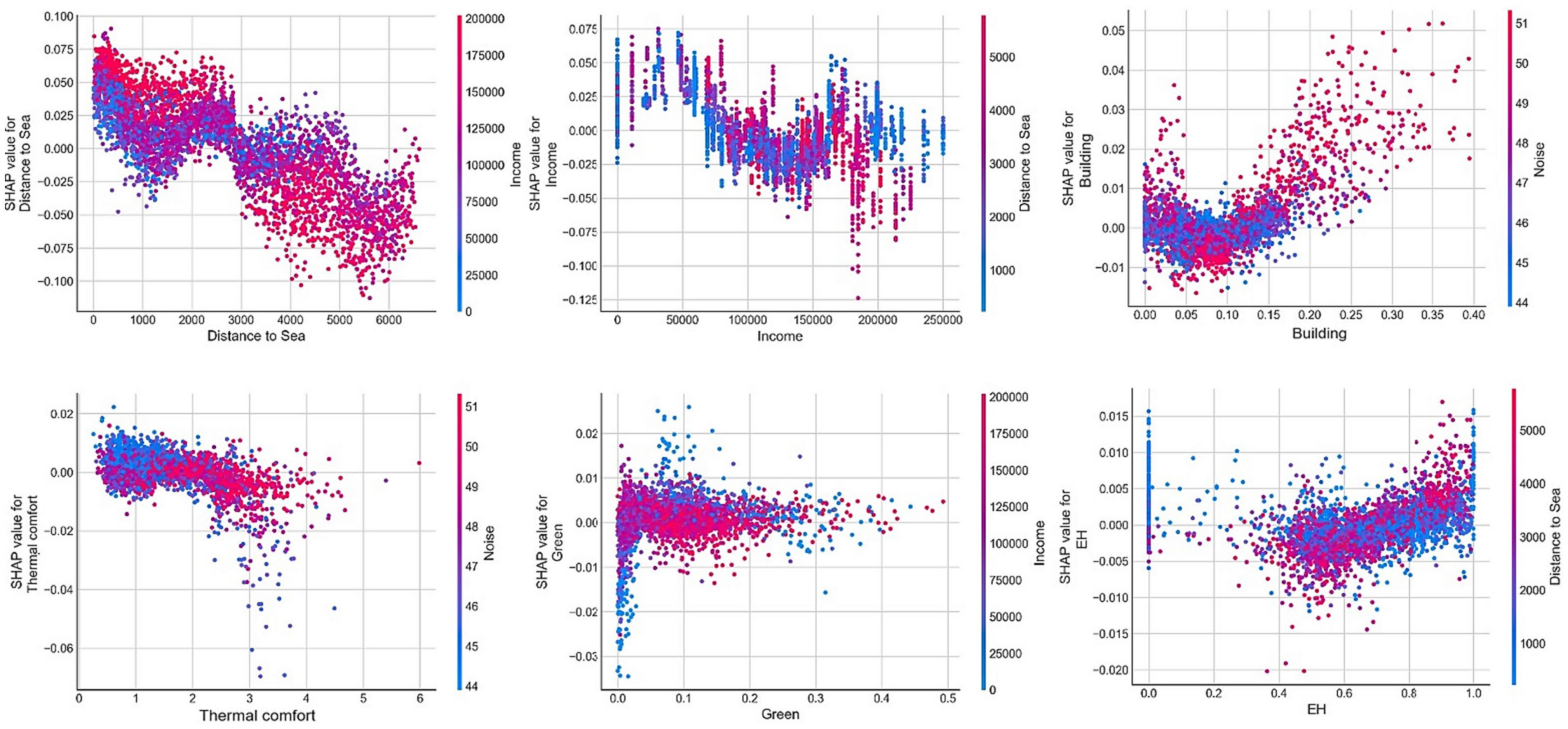
Local interaction effects among built environmental variables.

This section delves deeper into the local interaction effects among built environmental variables through the PDP analysis. We computed the pairwise interaction effects between the built environment variables using the absolute value of Shapley. Figure 10 displays the groups of built environment variables that have instructive interaction effects. In each graph of Figure 10, the X-axis represents the variable of interest, and the right Y-axis suggests the variable that has the strongest interaction effect with the variable of interest. The color of the dots corresponds with the value of the right Y-axis. Meanwhile, the positive and negative Shapley values on the left Y-axis indicate the correlation between the dependent and independent variables. A Shapley value greater than zero indicates a synergistic effect, jointly promoting the increase of the dependent variable, while a value less than zero indicates an antagonistic effect. The distance on the Y-axis represents the degree of significance of the correlation.

The distance to sea exhibits the strongest interaction with the income variable. In areas located within 3 km from the coastline, an increase in the residents’ income significantly promotes the increase of the sentiment index. This suggests that low-income individuals might have poorer access to the surrounding beaches and recreation places compared with high-income individuals, possibly due to time and energy constraints for enjoying holidays and entertainment among low-income groups. In terms of the building ratio index, a worth noting interaction effect is noise. When the building ratio index is between 0.04 and 0.1, and the noise levels are higher (above 49 dB), the two variables exhibit antagonism. However, when the building ratio index exceeds 0.2, the interaction between two variables turns into synergy. This may be because a low building ratio index can indicate a location in the suburbs or a high-quality low-density residential area where people are more sensitive to noise. In the central city area with a high building ratio index, people might get accustomed to noise and be less sensitive to it. Besides, high noise levels also occur in those vibrant city areas where various socioeconomic activities aggregate, thus promoting the generation of positive emotions. Furthermore, the synergistic effect between the thermal comfort index and the noise is strongest. Under appropriate thermal comfort conditions (the absolute difference from the comfortable temperature is less than 2°C) and a quiet acoustic environment (less than 47 dB), the two variables exhibit synergy. This finding aligns with existing research that indicates that a comfortable microenvironment contributes to physical and mental well-being (Nagano and Horikoshi, 2005), thereby stimulating positive emotions. Finally, the synergistic effect between land use diversity and the distance to sea is strongest. For areas far from the coastline (over 4 km), a synergistic effect can be observed when the land use diversity exceeds 0.7, while the opposite effect becomes antagonistic. This result is reasonable because areas far from recreational places tend to have less land use diversity. Higher land use diversity implies more diverse urban function and enables rich activities, thereby promoting positive emotions.

### 4.6 Hierarchical clustering analysis of sentiment index

**Figure 11:**
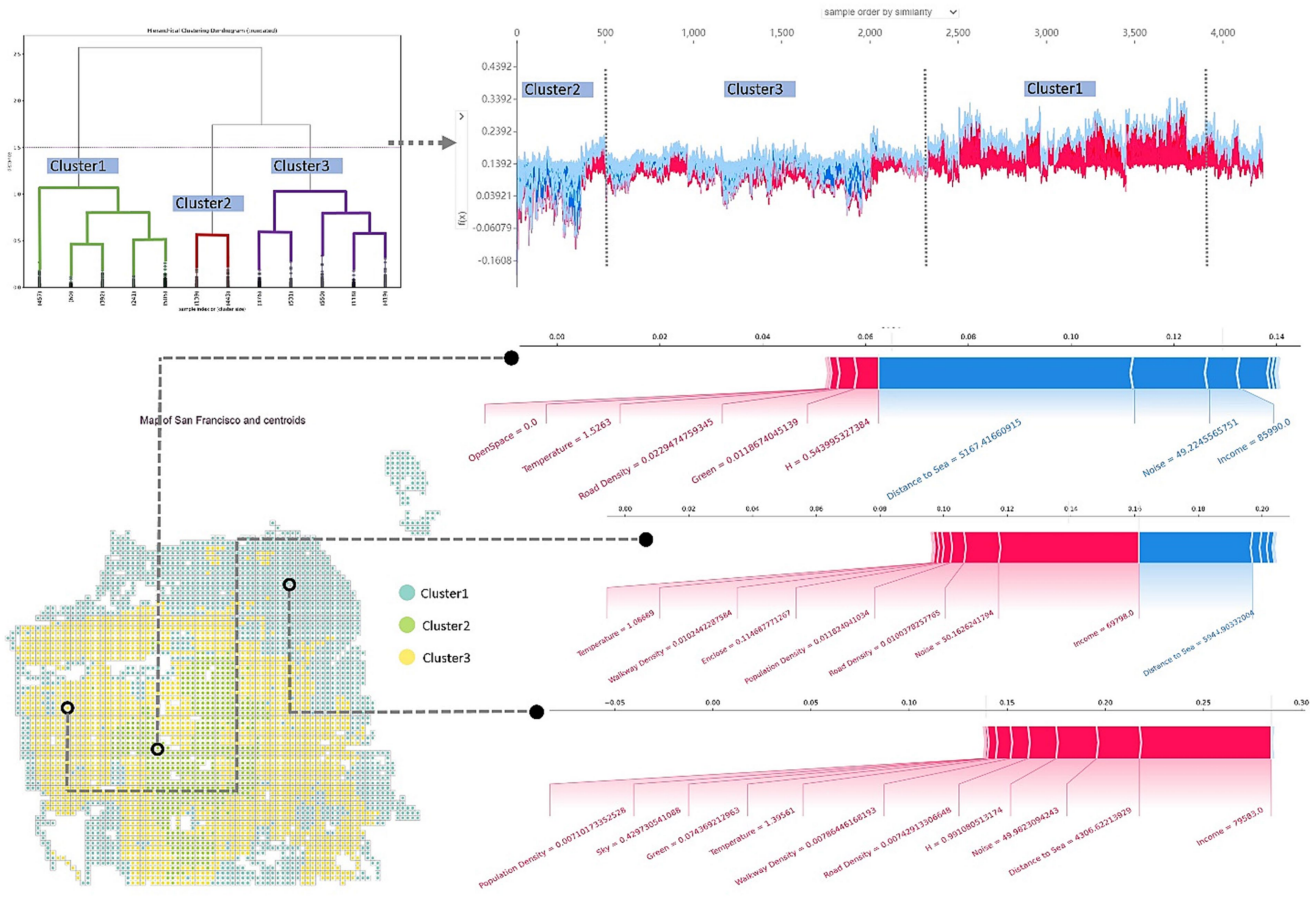
Sentiment index distribution clusters identified based on local effects.

The above-mentioned studies have demonstrated the complex relationship between the built environment and the semantic orientations of public emotion. Assessing the effect of built environmental variables on public emotion based on the Twitter sentiment analysis can help provide useful policy recommendations for promoting overall positive emotions at the city level. As previously mentioned, the distribution of the public sentiment index is determined by complex environmental factors, including spatial, environmental, and population differences. Hence, relatively general policy recommendations are not universal and cannot meet the detailed requirements of precisely promoting overall positive emotions in different regions. We conducted the hierarchical clustering analysis on the grid cells based on the synergistic effects of the environmental variables (SHAP values; Xiao et al., 2021). As a result, the grid cells were classified into three clusters according to the Elbow Method, in grid cells of the same cluster, the built environment variables have similar local effects on the sentiment index. Among the three clusters, Cluster 1 exhibits the highest sentiment index values, while Cluster 2 shows the lowest (Figure 11). As shown in the right part of Figure 11, the number of grid cells in three clusters is 1,655, 582, and 1994 respectively, and they are sorted on the X-axis according to similarity. Blue represents negative Shapley values, which inhibit the sentiment index. Red represents positive Shapley values, which increase the sentiment index. The lower left part of Figure 11 also shows the spatial distribution of the three clusters. Cluster 2, which has significantly more negative Shapley values among the three groups, is located in the middle of the city. Grid cells of Cluster 1 with more positive Shapley values mainly locate at the periphery area of the city and close to the coastline, while the grid cells of Cluster 3 locate between the Cluster 1 and 2. The three clusters also suggest an aggregation in spatial distribution as Figure 11 shows.

Given the different local effects of each cluster, targeted recommendations are necessary to promote positive emotions in the respective areas. In particular, Cluster 2 located in the central area has the lowest sentiment index among the three clusters and must be given priority consideration. The major environmental variables that decrease the sentiment index in Cluster 2 areas are the distance to sea and the noise. Meanwhile, the important variables that can effectively stimulate positive emotions in Cluster 2 areas are the land use diversity, the green ratio index, the road density, and the thermal comfort index. This result suggests that the low sentiment index in Cluster 2 areas may be affected by issues related to their distance from the coastline, parks, and other recreational areas. Moreover, the sensitivity to noise and insufficiency in land use diversity and greenery are also major issues that inhibit positive emotions. Considering the nonlinear relationship and synergistic effect, we can propose targeted policy recommendations to promote positive emotions in various urban areas. For instance, in the cluster 2 region characterized by the most negative emotions, initiatives such as constructing parks, lakes, and other recreational spaces should be implemented. Furthermore, efforts should be directed toward enhancing the regional acoustic environment and minimizing both traffic and construction noise. The current land use diversity should be increased from 0.54 to 0.8 or above, and the green ratio index should be increased from 0.011 to 0.07 or above to produce synergistic effects and promote positive emotion. Similarly, according to the local effects of environmental variables in Cluster 3 areas, income inequality should be reduced and population density should be increased, which will effectively help generate more positive emotions in the corresponding grid cells. Besides, the improvement in the micro-environment can also help Cluster 3 areas effectively promote positive emotions, such as increasing the green land ratio, reducing the sky ratio, and enriching the visual diversity of the environment.

## 5 Conclusion and discussion

This study quantifies the positiveness of public emotions through Tweet sentiment analysis and explores the spatial relationship between the urban environment and public emotions taking San Francisco as a study case. From macro- and micro-built environment perspectives, this study provides a new viewpoint for exploring the complex relationship between the semantic orientation of public emotions and the urban environment.

1. This paper addresses several key points. Compared to previous studies focusing on the macro-built environment, our research demonstrated that micro-built environmental factors have a significant impact on the semantic orientation of public emotions. For example, visual and auditory experience can affect human perception of an environment and have a greater impact on people’s feelings in an environment compared with temperature perception, which is consistent with some existing studies (Jiang et al., 2021). The finding emphasizes the importance of taking a micro-environment perspective when formulating policies to improve the urban environment. Traditional data collection methods, which primarily focus on the macro perspective, are not sufficient in capturing human perceptions of the urban environment. Nevertheless, with the advancement of technology and the widespread use of the internet, we now have access to abundant social data that allows us to directly understand how people feel about the urban environment. Therefore, this study suggests that urban planners and policymakers should consider the micro perspective based on human feelings in urban environments when designing strategies and initiatives to create better urban environments. This approach is crucial for achieving detailed urban governance and prioritizing public well-being.
2. Combining Random Forest (RF) and SHAP algorithms, this study confirmed the nonlinear influence of the built environmental factors on the polarity of public emotions. For instance, we observed a marginal effect in the relationship between residents’ proximity to the coastline and the sentiment index. As the distance from the coastline increases in residential areas, the sentiment index tends to decrease, signifying a more negative emotional state among residents–a trend that aligns with Brereton’s findings (Brereton et al., 2008). However, when this distance is less than 2.8 kilometers, the proximity to the coastline does not significantly impact the sentiment index. Namely, there is a noticeable threshold effect of urban environments on the promotion of public positive emotions. This result is instructive for it helps policymakers to choose the most effective environment improvement decision under finite financial budget based on the impact strength and usefulness range of different environment aspects.
3. This study discovered various synergistic effects among built environmental variables, thereby further revealing the intricate relationship between the built environment factors and public emotions. There is evident inequality in the promoting effects of built environment factors on public positive emotions, such as heightened sensitivity to noise among residents in suburban areas with lower building density. Moreover, this study also addressed various synergic relationships between urban environmental factors and asserted that considering the synergies between certain variables can maximize the promotion of positive emotions among the public. For instance, an appropriate thermal comfort environment (with an absolute temperature difference less than 2) and suitable acoustic conditions (with decibel levels less than 47) have the greatest impact on fostering positive emotions.

Accordingly, this study proposed a fine-grained urban environment governance approach that formulates customized strategies for promoting positive emotions in regions with different local synergistic effects (i.e., clustering based on SHAP values). Unlike those one-size-fits-all strategies, this approach enables urban planners and policymakers to consider the inequality in environmental perceptions and the interactions between environmental factors. For example, in the relative importance ranking in Section 4.3, we found that for the entire city of San Francisco, income ranked second in its impact on public emotions. However, in the hierarchical clustering analysis in Section 4.6, we discovered that in cluster 2, where public emotions were most negative, the impact of noise was more significant than income. We believe that this will aid in formulating more efficient and targeted strategies for urban improvement, thus holding practical significance in effectively enhancing residents’ well-being.

However, this study also has certain limitations. First, this study only focuses on the public emotion distribution in San Francisco, which is surrounded by three seas and has a pleasant climate, and the relationship between the environmental variables and the sentiment index may not necessarily be transferrable to other cities. For example, the distance to sea and the thermal comfort, which have already suggested an inconsistency with previous studies. Future research can explore the emotion distribution and its influencing factors in different types of cities. Second, the greater the size of the tweet data, the more valuable the results may be obtained. More samples (such as more than a few million data) may implicitly suggest deeper influencing relationships, such as spatiotemporal distribution differences in emotion among residents of varying genders, ages, and races. Finally, this study focuses on the nonlinear influence of micro- and macro-built environmental factors on the semantic orientations of public emotions and the synergistic effect between variables, paying limited attention to the spatial heterogeneity of emotion distribution. Despite the shortcomings, we hope that this empirical study can provide references and suggestions for relevant policymakers to reduce inequality and negative sentiment and achieve healthy and sustainable cities.

## Data availability statement

The original contributions presented in the study are included in the article/supplementary material, further inquiries can be directed to the corresponding authors.

## Author contributions

PH: Conceptualization, Formal analysis, Investigation, Software, Supervision, Supervision, Writing – review & editing. BY: Conceptualization, Data curation, Investigation, Methodology, Writing – review & editing. JM: Methodology, Software, Writing – original draft, Writing – review & editing. KL: Investigation, Methodology, Software, Writing – review & editing. SC: Conceptualization, Data curation, Formal analysis, Funding acquisition, Resources, Software, Writing – review & editing. ZS: Conceptualization, Data curation, Funding acquisition, Writing – original draft, Writing – review & editing.

## Footnotes

^1^ https://datasf.org/opendata/
